# Food intake in children and adolescents: a cross-sectional study from 2009/2010 to 2015/2016

**DOI:** 10.1017/jns.2021.40

**Published:** 2021-07-15

**Authors:** Maria N. Tell, Katarina Hedin, Mats Nilsson, Marie Golsäter, Hans Lingfors

**Affiliations:** 1 Futurum, Region Jönköping County, and Department of Health, Medicine and Caring Sciences, Linköping University, Jönköping, Sweden; 2 Department of Clinical Sciences in Malmö, Family Medicine, Lund University, Lund, Sweden; 3 Child Research Group, Department of Nursing, School of Health and Welfare, Jönköping University, Jönköping, Sweden

**Keywords:** Adolescents, Children, Eating habits, Food intake, Time trend, IHME, Institute for Health Metrics and Evaluation, NNR, Nordic Nutrition Recommendations, SSBs, sugar-sweetened beverages

## Abstract

The aim was to describe food intake over time in children and adolescents, with respect to age and gender. The present study was a repeated cross-sectional study using self-reported data from a health questionnaire. Data were collected from the School Health Services in south-east Sweden from 44 297 students, 10, 13–14 and 16 years of age, 48 % girls and 52 % boys from 2009/2010 to 2015/2016. Reported intakes for eight foods were analysed in relation to the Nordic Nutrition Recommendations. Seventeen per cent of the students reported an intake in line with the recommendations for at least six of the eight foods fish, vegetables, fruit, mealtime beverages, juice/chocolate drinks, sugar-sweetened beverages, sweets/snacks and pastries. Intake of at least two fruits a day was the recommendation that was followed by the lowest proportion of students (30 %), and this result was stable over the study period. There was a gradual increase over time in the proportion of students who reported an intake in line with the recommendations. Younger students compared to older students, as well as girls compared to boys, reported intakes in line with the recommendations to a statistically significant larger extent. Few students reported intakes in line with the recommendations, although the quality of food intake seems to improve over time. The present results indicate a deterioration in reported food intake in the early years of adolescence. Finding methods to support all children and especially older adolescents to eat healthier, including eating more fruit, is of great importance to public health.

## Introduction

A healthy diet is associated with lower mortality, independent of other lifestyle factors^(1)^. According to the Institute for Health Metrics and Evaluation (IHME), poor diet is one of the top risk factors for all-cause mortality^(2,3)^. Even in childhood, diet can influence the atherosclerotic process, and dietary patterns may predict cardiovascular disease^(4)^. Food habits established early in life may also have an influence on food habits in adulthood^(5–7)^. For example, a high intake of fruit in childhood has been shown to be associated with a lower incidence of cancer in adulthood^(8)^. Few adolescents in Western countries eat the recommended amount of fruit and vegetables^(9,10)^, although girls, in general, have a healthier dietary pattern than boys^(11)^.

The food advice from the Swedish Food Agency is based on the Nordic Nutrition Recommendations (NNR) issued by the Nordic Council of Ministers^(12,13)^, and these recommendations resemble the Mediterranean diet^(14)^. The food advice means that children aged 10 and over should eat at least 500 g of fruit and vegetables a day, whole grain products rather than processed carbohydrates, fish at least twice a week and unsaturated rather than saturated fats^(15,16)^.

A few reports have indicated a deterioration in eating habits during the transition from childhood to adolescence^(17,18)^. This deterioration has, however, not been sufficiently investigated. Only a few recently published international studies have described food intake time trends among children and adolescents^(9)^. During the transition from childhood to adolescence, the school and the School Health Services are important arenas for health promotion. To further explore break points in the deterioration in eating habits, data from the School Health Services provides a unique possibility to describe food intake over time.

The aim of the present study is to describe self-reported food intake over time in children and adolescents, with respect to age and gender.

## Methods

### Study design

The present study is a repeated cross-sectional study in seven cohorts of children and adolescents.

### Setting

In Sweden, all students in primary schools are provided with nutritious and free school lunches^(19)^. The Swedish Education Act further stipulates that four health visits, including a health dialogue concerning health and lifestyle, should be offered by the School Health Services to all children and adolescents during their school years^(19,20)^. In Jönköping County in south-east Sweden, the setting of the present study, a questionnaire called ‘My Health’, is used as a basis in the structured health dialogue^(21)^. The studied schools corresponded to about half of the children and adolescents in Jönköping County during the academic years 2009/2010 up to 2015/2016^(22)^. The children and adolescents complete the ‘My Health’ questionnaire in writing, with support from the school nurse who is present in the classroom. The answers to the questionnaire can also be adjusted during the health dialogue.

By means of the questionnaire, the 10 year olds answer eight questions about food intake, and the adolescents also two additional questions. The eight common questions for all age groups relate to intake of fish, vegetables, fruit, mealtime beverages, juice/chocolate drinks, sugar-sweetened beverages (SSBs), sweets/snacks and pastries. The two additional questions aimed at the adolescents concerning butter/margarine as sandwich spread and sandwich toppings. For most of the questions, the students are asked to state how frequently they consumed the stated food during the last 7 d, with four to seven response alternatives (Table 1).

**Table 1.** Responses to the ‘My Health’ questionnaire among 44 297 students aged 10, 13–14 and 16 in the academic years 2009/2010 up to 2015/2016.

Questions if you think back to the latest 7 d, …	Response alternatives	10 (years)		13–14 (years)		16 (years)	
		Girls, *n* (%)	Boys, *n* (%)	Girls, *n* (%)	Boys, *n* (%)	Girls, *n* (%)	Boys, *n* (%)
… how often did you have fish?	At least 3 times/week^a^	1 323 (17)^b^	1 486 (18)^b^	530 (8)^c^	734 (10)^c^	355 (6)^c^	471 (7)^c^
	Twice/week^a^	2 431 (31)	2 256 (27)	2 351 (33)	2 560 (34)	1 921 (31)	2 483 (35)
	Once/week	2 915 (37)	3 004 (36)	2 921 (42)	3 019 (40)	2 686 (43)	3 004 (42)
	Never	1 174 (15)	1 542 (18)	1 217 (17)	1 284 (17)	1 282 (21)	1 172 (16)
… how often did you have vegetables?	At least 3 times/day^a^	1 629 (21)^b^	1 870 (22)^b^	597 (8)^b^	714 (9)^b^	390 (6)^b^	379 (5)^b^
	About twice/day^a^	2 923 (37)	2 473 (30)	2 279 (32)	1 848 (24)	1 938 (31)	1 451 (20)
	About once/day	2 001 (25)	1 990 (24)	2 126 (30)	2 186 (29)	1 948 (31)	2 145 (30)
	5–6 times/week	666 (8)	770 (9)	655 (9)	748 (10)	617 (10)	813 (11)
	3–4 times/week	329 (4)	533 (6)	715 (10)	960 (13)	697 (11)	1 094 (15)
	1–2 times/week	250 (3)	470 (6)	506 (7)	813 (11)	504 (8)	922 (13)
	Never	78 (1)	213 (3)	146 (2)	331 (4)	149 (2)	338 (5)
… how many fruits did you have?	At least 3 fruits/day^a^	1 144 (15)^b^	1 324 (16)^b^	457 (6)	557 (7)	350 (6)^b^	315 (4)^b^
	About 2 fruits/day^a^	2 795 (35)	2 179 (26)	1 255 (18)	1 199 (16)	1 020 (16)	826 (12)
	About 1 fruit/day	2 424 (31)	2 435 (29)	2 161 (31)	1 991 (26)	1 682 (27)	1 588 (22)
	About 3–4 fruits/week	648 (8)	809 (10)	1 405 (20)	1 443 (19)	1 374 (22)	1 492 (21)
	About 1–2 fruits/week	694 (9)	1 179 (14)	1 331 (19)	1 673 (22)	1 337 (21)	1 890 (26)
	No fruit	167 (2)	394 (5)	413 (6)	739 (10)	480 (8)	1 030 (14)
… what mealtime beverages did you usually have?	Water^a^	3 810 (48)^b^	3 272 (39)^b^	3 598 (51)^b^	2 782 (36)^b^	3 330 (53)^b^	2 230 (31)^b^
	Milk (0⋅5 % fat)^a^	1 133 (14)	1 263 (15)	845 (12)	1 057 (14)	701 (11)	921 (13)
	Milk (1⋅5 % fat)	2 302 (29)	2 777 (33)	1 956 (28)	2 692 (35)	1 448 (23)	2 587 (36)
	Milk (3⋅0 % fat)	302 (4)	440 (5)	251 (4)	516 (7)	235 (4)	618 (9)
	Soda or juice	312 (4)	549 (7)	366 (5)	548 (7)	526 (8)	782 (11)
… how often did you have at least one glass of chocolate drink or juice?	Never^a^	1 183 (15)^b^	1 206 (14)^b^	1 466 (21)^b^	1 419 (19)^b^	1 573 (25)^b^	1 706 (24)^b^
	Once/week^a^	2 763 (35)	2 787 (33)	1 926 (27)	1 913 (25)	1 438 (23)	1 466 (20)
	2–3 times/week	2 458 (31)	2 371 (28)	2 083 (30)	2 284 (30)	1 800 (29)	1 953 (27)
	4–5 times/week	698 (9)	922 (11)	791 (11)	970 (13)	661 (11)	911 (13)
	Almost every day	637 (8)	783 (9)	647 (9)	853 (11)	661 (11)	894 (12)
	Several times/day	115 (1)	227 (3)	110 (2)	170 (2)	107 (2)	202 (3)
… how often did you have at least one glass of soft drink, energy/sports drink (SSBs)?	Never^a^	978 (12)^b^	842 (10)^b^	911 (13)^b^	603 (8)^b^	1 006 (16)^b^	514 (7)^b^
	Once/week^a^	3 220 (41)	2 902 (35)	2 158 (31)	1 836 (24)	1 693 (27)	1 193 (17)
	2–3 times/week	2 982 (38)	3 312 (40)	3 095 (44)	3 624 (48)	2 467 (39)	3 158 (44)
	4–5 times/week	424 (5)	770 (9)	594 (8)	1 043 (14)	661 (11)	1 382 (19)
	Almost every day	180 (2)	301 (4)	198 (3)	351 (5)	302 (5)	656 (9)
	Several times/day	66 (0⋅8)	166 (2)	63 (0⋅9)	150 (2)	108 (2)	233 (3)
… how often did you have sweets or snacks (chocolate, ice cream, sweets, chips)?	Never^a^	340 (4)^b^	376 (5)^b^	427 (6)	412 (5)	443 (7)^c^	549 (8)^c^
	Once/week^a^	3 612 (48)	3 295 (39)	2 205 (31)	2 357 (31)	1 614 (26)	1 939 (27)
	2–3 times/week	3 463 (44)	3 893 (47)	3 655 (52)	3 968 (52)	3 148 (50)	3 498 (49)
	4–5 times/week	314 (4)	505 (6)	559 (8)	638 (8)	698 (11)	795 (11)
	Almost every day	74 (0⋅9)	140 (2)	146 (2)	176 (2)	288 (5)	295 (4)
	Several times/day	47 (0⋅6)	80 (1)	34 (0⋅5)	58 (0⋅8)	50 (0⋅8)	64 (0⋅9)
… how often did you have cookies, biscuits, buns or cakes (pastries)?	Never^a^	1 026 (13)^b^	1 085 (13)^b^	1 387 (20)	1 577 (21)	1 421 (23)	1 683 (24)
	Once/week^a^	3 834 (49)	3 638(44)	3 019 (43)	3067 (4)	2 347 (38)	2 536 (35)
	2–3 times/week	2 446 (31)	2 653 (32)	2 137 (30)	2 310 (30)	1 937 (31)	2 184 (31)
	4–5 times/week	399 (5)	609 (7)	326 (5)	456 (6)	379 (6)	503 (7)
	Almost every day	118 (2)	233 (3)	136 (2)	145 (2)	128 (2)	199 (3)
	Several times/day	26 (0⋅3)	75 (0⋅9)	18 (0⋅3)	48 (0⋅6)	27 (0⋅4)	32 (0⋅4)

The *P*-values of <0⋅05 are considered statistically significant when comparing proportions of boys and girls who reported food intakes in line with the NNR. Missing values are no more than 1 % for any question and are not shown in the table.

a Intakes of foods in line with the NNR.

b A statistically significantly higher proportion (*P* < 0⋅05) of girls reported a food intake in line with the NNR.

c A statistically significantly higher proportion (*P* < 0⋅05) of boys reported a food intake in line with the NNR.

### Study population

**Fig. 1.**
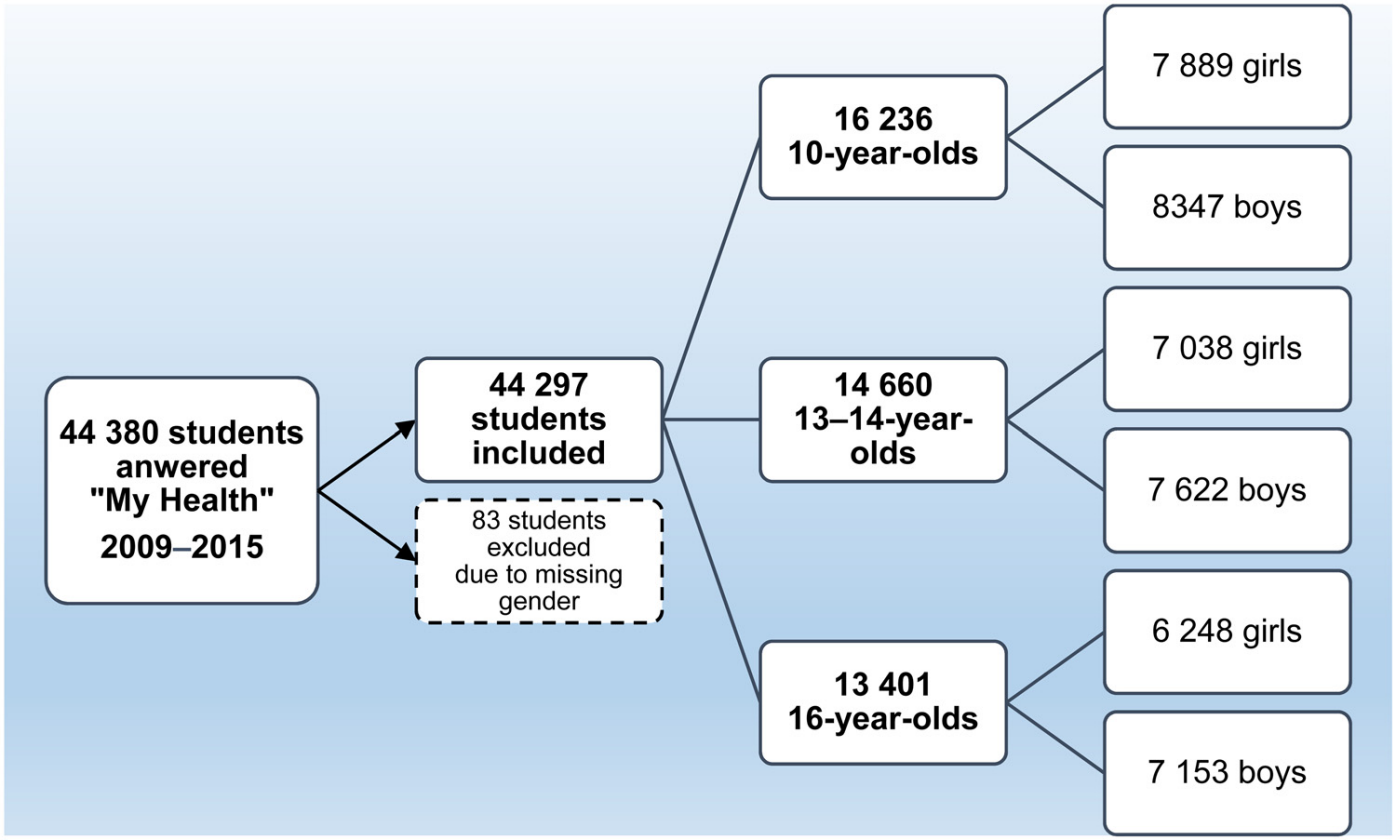
Flow chart of included students.

The study population consisted of seven cohorts of children and adolescents who turned 10, 13–14 and 16 during the academic years 2009/2010 up to 2015/2016 in Jönköping County in south-east Sweden. All students were invited to the health dialogue, and almost all agreed to take part. Of the 44 380 students who responded to the questionnaire, 0⋅2 % with missing gender information were excluded, resulting in 44 297 included students (Fig. 1). The proportions of missing values for specific questions in the questionnaires varied between 0 and 1 %.

### Statistical analyses

To describe and compare food intake, the responses to the questions were classified according to how many of the reported intakes were in line with the recommendations (Table 1), based on the NNR^(12,13)^ and the Swedish Food Agency^(15,16)^. Descriptive statistics were used to describe the categorical variables using numbers and percentages. Differences in distribution between groups for categorical variables were tested with likelihood-ratio *χ*^2^, and for time trend, the Mantel–Haenszel *χ*^2^ test was used. A *P*-value <0⋅05 was considered statistically significant. We used Benjamin-Hochberg's method to correct for multiple comparisons^(23)^.

## Results

The overall intake frequencies from 2009/2010 to 2015/2016 in students (children and adolescents) are presented by gender and age group in Table 1.

### Adherence to the recommendations

**Fig. 2.**
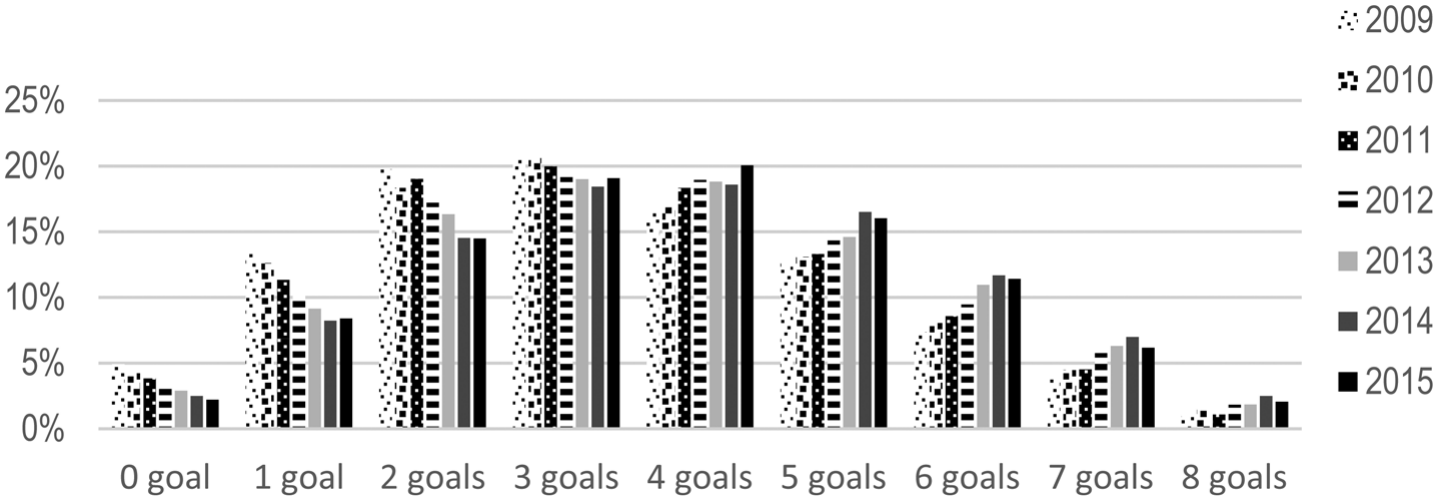
Proportions of students (*n* 44 297), aged 10, 13–14 and 16 years, who reported an intake in line with the NNR for zero to eight of the foods, between the academic years 2009/2010 up to 2015/2016.

Overall, 31 % of the students reported an intake in line with the recommendations for up to two of the eight foods, 38 % three to four foods, 24 % five or six foods and 7 % seven or eight foods. Seventeen per cent of the students reported an intake in line with the recommendations for at least six of the eight foods (Table 2). Significantly more students (*P* < 0⋅0001) reported an intake in line with the recommendations at the end of the study (2015/2016), when 20 % reported an intake in line with the recommendations for six to eight of the foods, compared with the start of the study (2009/2010), when 12 % reported an intake in line with the recommendations for six to eight of the foods (Fig. 2).

**Table 2.** Number and proportions of students^a^ aged 10, 13–14 and 16 in the academic years 2009/2010 up to 2015/2016, who reported food intakes in line with the NNR for zero to eight foods

Number of foods in line with the recommendations (*n*)	Girls (years)			Boys (years)			All girls *n* (%)	All boys *n* (%)	All students *n* (%)
	10 *n* (%)	13–14 *n* (%)	16 *n* (%)	10 *n* (%)	13–14 *n* (%)	16 *n* (%)			
0	108 (1)	193 (3)	207 (3)	236 (3)	321 (4)	432 (6)	508 (2)	989 (4)	1 497 (3)
1	414 (5)	651 (9)	667 (11)	702 (8)	1 036 (14)	1 155 (16)	1 732 (8)	2 893 (13)	4 625 (10)
2	917 (12)	1 197 (17)	1 149 (18)	1 219 (15)	1 430 (19)	1 645 (23)	3 263 (15)	4 294 (19)	7 557 (17)
3	1 262 (16)	1 422 (20)	1 312 (21)	1 503 (18)	1 647 (22)	1 514 (21)	3 996 (19)	4 664 (20)	8 660 (20)
4	1 497 (19)	1 387 (20)	1 159 (19)	1 573 (19)	1 344 (18)	1 159 (16)	4 043 (19)	4 076 (18)	8 119 (18)
5	1 390 (18)	1 081 (15)	903 (14)	1 327 (16)	981 (13)	688 (10)	3 374 (16)	2 996 (13)	6 370 (14)
6	1 198 (15)	664 (9)	541 (9)	984 (12)	538 (7)	365 (5)	2 403 (11)	1 887 (8)	4 290 (10)
7	824 (10)	341 (5)	238 (4)	602 (7)	255 (3)	151 (2)	1 403 (7)	1 008 (4)	2 411 (5)
8	277 (4)	97 (1)	72 (1)	195 (2)	63 (1)	44 (1)	446 (2)	302 (1)	748 (2)

a Twenty of the students did not answer any of the questions concerning food intake.

The younger students reported an intake in line with the recommendations for significantly more foods than the older ones (*P* < 0⋅0001). Twenty-five per cent of the 10 year olds reported an intake in line with the recommendations for at least six of the eight foods, compared with 13 % of 13–14 year olds and 11 % of 16 year olds (Table 2).

Girls reported an intake in line with the recommendations for significantly more foods compared with boys (*P* < 0⋅0001). In the group of 10 year olds, 29 % of girls reported an intake in line with the recommendations for six to eight of the foods, compared with 21 % of boys. The corresponding figures for the 13–14 year olds were 16 % for girls and 11 % for boys. Among the 16 year olds, 14 % of girls and 8 % of boys reported an intake in line with the recommendations for six to eight of the foods (Table 2).

### Adherence to the recommendations for separate foods

The proportions of students reporting an intake in line with the recommendations for the separate foods for each of the separate years, taking gender and age groups into consideration, are presented in Table 3 (girls) and Table 4 (boys). Over the study period, the reported intake became healthier in terms of increased proportions of students reporting an intake in line with the recommendations for the separate foods. With the exception of fruit intake, the healthy time trends were statistically significant (*P* < 0⋅0001) for all foods, for the three age groups together. Fruit intake was stable over time in all age and gender groups (Tables 3 and 4).

**Table 3.** Number and proportions of girls aged 10, 13–14 and 16 in the academic years 2009/2010 up to 2015/2016, who reported food intakes in line with the NNR

Foods in line with the recommendations	Age group (years)	Year: 2009/2010 *n* (%)	Year: 2010/2011 *n* (%)	Year: 2011/2012 *n* (%)	Year: 2012/2013 *n* (%)	Year: 2013/2014 *n* (%)	Year: 2014/2015 *n* (%)	Year: 2015/2016 n (%)	Year: 2009/2010–2015/2016 *n* (%)
Fish Twice/week or more	10	375 (48)	504 (41)	555 (49)	588 (51)	522 (49)	600 (48)	610 (50)	3 754 (48)
	13–14	350 (39)	305 (39)	535 (41)	375 (39)	398 (43)	430 (41)	488 (43)	2 881 (41)
	16	307 (32)	382 (34)	388 (37)	279 (40)	239 (39)	285 (37)	396 (39)	2 276 (36)
Vegetables Twice/day or more	10	432 (55)	679 (54)	665 (58)	675 (58)	638 (60)	744 (60)	719 (58)	4 552 (58)
	13–14	319 (36)	295 (37)	524 (41)	346 (36)	359 (39)	481 (46)	552 (49)	2 876 (41)
	16	323 (33)	373 (33)	371 (35)	267 (38)	252 (41)	324 (42)	418 (42)	2 328 (37)
Fruit Two/day or more	10	379 (49)	640 (51)	602 (53)	580 (50)	566 (53)	615 (49)	575 (45)	3 939 (50)
	13–14	231 (26)	224 (28)	296 (23)	199 (21)	227 (25)	260 (25)	275 (24)	1 712 (24)
	16	209 (22)	231 (21)	229 (22)	145 (21)	161 (26)	181 (24)	214 (21)	1 370 (22)
Mealtime beverages Water and milk (0⋅5 % fat)	10	410 (53)	719 (58)	680 (60)	703 (61)	749 (70)	866 (70)	816 (66)	4 943 (63)
	13–14	521 (58)	474 (60)	768 (60)	582 (60)	614 (67)	716 (69)	768 (68)	4 443 (63)
	16	597 (62)	669 (59)	618 (59)	465 (66)	407 (66)	573 (74)	702 (70)	4 031 (65)
Juice/chocolate drinks Once/week or less	10	368 (47)	558 (45)	532 (47)	566 (49)	527 (49)	708 (57)	687 (56)	3 946 (50)
	13–14	385 (43)	351 (44)	596 (46)	407 (42)	462 (50)	534 (51)	657 (58)	3 392 (48)
	16	406 (42)	507 (45)	473 (45)	330 (47)	326 (53)	431 (56)	538 (53)	3 011 (48)
SSBs Once/week or less	10	377 (49)	618 (50)	567 (50)	593 (52)	582 (55)	730 (59)	731 (59)	4 198 (53)
	13–14	350 (39)	349 (44)	510 (40)	390 (41)	406 (44)	511 (49)	553 (49)	3 069 (44)
	16	402 (42)	455 (40)	411 (39)	303 (43)	294 (48)	373 (48)	461 (46)	2 699 (43)
Sweets/snacks Once/week or less	10	423 (54)	633 (51)	575 (51)	570 (50)	540 (51)	630 (51)	581 (47)	3 952 (50)
	13–14	326 (36)	307 (39)	452 (35)	363 (38)	335 (36)	425 (41)	424 (38)	2 632 (37)
	16	323 (33)	351 (31)	335 (32)	232 (33)	214 (35)	270 (35)	332 (33)	2 057 (33)
Pastries Once/week or less	10	444 (57)	703 (57)	690 (61)	706 (61)	662 (62)	816 (65)	839 (68)	4 860 (62)
	13–14	517 (58)	470 (59)	766 (59)	600 (62)	593 (64)	680 (65)	780 (69)	4 406 (63)
	16	575 (59)	657 (58)	624 (59)	407 (58)	367 (59)	487 (63)	651 (65)	3 768 (60)

**Table 4.** Number and proportions of boys aged 10, 13–14 and 16 in the academic years 2009/2010 up to 2015/2016, who reported food intakes in line with the NNR

Foods in line with the recommendations	Age group (years)	Year: 2009/2010 *n* (%)	Year: 2010/2011 *n* (%)	Year: 2011/2012 *n* (%)	Year: 2012/2013 *n* (%)	Year: 2013/2014 *n* (%)	Year: 2014/2015 *n* (%)	Year: 2015/2016 *n* (%)	Year: 2009/2010–2015/2016 *n* (%)
Fish Twice/week or more	10	360 (43)	543 (42)	506 (42)	557(46)	545 (48)	578 (45)	653 (49)	3 742 (45)
	13–14	414 (43)	364 (39)	585 (43)	474 (44)	407 (43)	484 (45)	566 (46)	3 294 (43)
	16	402 (37)	428 (39)	493 (40)	392 (44)	364 (42)	413 (46)	462 (42)	2 954 (41)
Vegetables Twice/day or more	10	383 (45)	633 (49)	618 (52)	656 (54)	620 (54)	707 (55)	726 (54)	4 343 (52)
	13–14	274 (29)	296 (32)	452 (33)	386 (36)	313 (33)	398 (37)	443 (36)	2 562 (34)
	16	238 (22)	276 (25)	283 (23)	241 (27)	227 (26)	251 (28)	314 (29)	1 830 (26)
Fruit Two/day or more	10	336 (40)	549 (43)	494 (41)	550 (45)	520 (46)	541 (42)	513 (38)	3 503 (42)
	13–14	215 (22)	226 (24)	307 (22)	245 (23)	225 (24)	257 (24)	281 (23)	1 756 (23)
	16	170 (16)	183 (17)	170 (14)	148 (17)	126 (15)	147 (16)	197 (18)	1 141 (16)
Mealtime beverages Water and milk (0⋅5 % fat)	10	377 (45)	629 (49)	590 (49)	658 (55)	657 (58)	841 (65)	783 (58)	4 535 (55)
	13–14	397 (42)	421 (45)	661 (48)	543 (50)	529 (56)	624 (58)	664 (54)	3 839 (51)
	16	406 (38)	436 (40)	502 (41)	411 (46)	368 (42)	486 (54)	542 (50)	3 151 (44)
Juice/chocolate drinks Once/week or less	10	374 (44)	536 (42)	535 (45)	594 (49)	547 (48)	667 (52)	740 (55)	3 993 (48)
	13–14	358 (37)	357 (38)	588 (43)	477 (44)	413 (43)	525 (49)	614 (50)	3 332 (44)
	16	424 (39)	418 (38)	507 (42)	418 (47)	414 (48)	446 (49)	545 (50)	3 172 (44)
SSBs Once/week or less	10	374 (44)	509 (40)	506 (42)	540 (45)	506 (45)	614 (47)	695 (52)	3 744 (45)
	13–14	244 (25)	255 (27)	422 (31)	354 (33)	327 (34)	392 (36)	445 (36)	2 439 (32)
	16	205 (19)	238 (22)	252 (21)	245 (28)	227 (26)	253 (28)	287 (26)	1 707 (24)
Sweets/snacks Once/week or less	10	377 (45)	568 (45)	530 (44)	537 (45)	503 (44)	574 (44)	582 (43)	3 671 (44)
	13–14	301 (31)	301 (32)	503 (37)	409 (38)	372 (39)	441 (41)	442 (36)	2 769 (36)
	16	292 (27)	349 (32)	409 (34)	345 (39)	346 (40)	331 (37)	416 (38)	2 488 (35)
Pastries Once/week or less	10	442 (52)	666 (52)	666 (56)	663 (55)	657 (58)	771 (60)	858 (64)	4 723 (57)
	13–14	535 (56)	551 (59)	795 (58)	688 (63)	565 (59)	714 (66)	796 (65)	4 644 (61)
	16	576 (53)	624 (57)	698 (57)	551 (62)	547 (63)	544 (60)	679 (62)	4 219 (59)

Over the study period, the proportion of 13–14-year-old and 16-year-old boys reporting an intake in line with the recommendations for intake of sweets/snacks increased, from 31 to 36 % (*P* < 0⋅0001) and from 27 to 38 % (*P* < 0⋅0001), respectively, while the intake of sweets/snacks among the 10-year-old boys was stable over time (Table 4). By contrast, seven percentage points fewer of the 10-year-old girls reported an intake in line with the recommendations for sweets/snacks at the study end (*P* 0⋅01), while 13–14 and 16-year-old girls had an unchanged intake over time (Table 3).

Younger students reported a healthier intake for all foods in the questionnaire compared to older students, with a few exceptions (see Tables 1, 3 and 4). The greatest differences were seen between 10 and 13–14 years of age. Girls reported a significantly healthier intake of the eight separate foods than boys (*P* < 0⋅0001), with the exception of fish (*P* 0⋅01). When analysing the three age groups separately, a similar result was seen, with a significantly higher proportion of girls than boys reporting an intake in line with the recommendations for the foods, with the exception of fish intake in the two older age groups and sweets/snacks among 16 year olds. There were no significant gender differences regarding intake of fruit, sweets/snacks and pastries among 13–14 year olds or pastries among 16 year olds (Table 1).

## Discussion

In this cross-sectional study, only a small group of students reported an overall healthy food intake over the 7-year period. Over time, the proportion of students reporting a healthy intake increased for most foods, with the exception of fruit and sweets/snacks. Younger students reported a healthier food intake than older students and girls reported a healthier food intake than boys, with a few exceptions.

While the present results show that only a few students had an overall healthy food intake, a healthy time trend can be noted. During the 7-year study period, changes in a healthier direction were seen for most foods at a group level. Only three out of ten students reported an intake in line with the recommendations for fruit, and this was stable over the whole study period. This is an exception to the trend of improved food intake over the study period, as a healthier intake of all the other studied foods was reported over the years. According to the IHME's Global Burden of Disease, low intake of whole grains, as well as low intake of nuts and seeds and low fruit intake, are leading dietary risk factors for death from non-communicable diseases, both in Sweden and globally^(24)^. The diversity of vegetables has been broadened in Swedish grocery stores in recent years^(25)^, and the consumption of vegetables has clearly increased for the whole Swedish population. This is in contrast to the consumption of fruit between 2010 and 2015, according to a report from Statistics Sweden^(26)^. The question of why fruit intake is low and stable, in the present study, in contrast to intake of vegetables, might be answered by looking at the way these foods are consumed during the day. In the Nordic countries, vegetables are often part of a complete meal, while fruits are mainly consumed between meals. Fruit intake may therefore be a more individual and conscious choice that has to be planned for.

In the present study, a significant difference was also found for a healthy mealtime beverage (water or low-fat milk), with an increased intake reported over time. This is in line with a report from Statistics Sweden^(26)^, which shows a decrease in milk consumption in the whole Swedish population. The number of students in the present study who had juice/chocolate drinks, SSBs, and pastries no more than once a week increased over time. This is in contrast to the aforementioned report, showing that the consumption of fruit juice as well as soda has increased in the Swedish population. However, the question in ‘My Health’ includes chocolate drinks, unlike the report from Statistics Sweden, which instead includes mineral water with soda/soft drinks. This means that the two results are not entirely comparable^(26)^.

An interesting exception to the deterioration with age concerning reported food choices among boys in the present study was the intake of sweets/snacks. The consumption of sweets/snacks among 10-year-old boys stayed at the same level throughout the study period, while a larger part of the boys in the two older groups reported an intake in line with the recommendations for sweets and snacks by the end of the study.

The finding that younger students generally report a healthier food intake than older students may be explained by younger individuals having fewer opportunities to make their own decisions about the type of food they eat. It is possible that children tend to follow their parents’ and other authorities’ example and advice more during the pre-pubescent period than in later adolescence. In addition, increased age brings more opportunities to choose one's own way of life (e.g. with more spending money available), and it can be hard to handle these opportunities responsibly in adolescence. The understanding of consequences grows with age and experience^(27)^, and this can be reflected in eating habits, where diet seems to become healthier after the early adult years^(28)^. To see if children or adolescents really preserve their habits into adulthood, we need to follow individual data. Other studies have shown that dietary patterns persist to some degree over time^(6)^. In the present study, the deterioration in food intake in early adolescence seemed to apply to the majority of the food groups.

In line with other studies^(29)^, the present study showed a gender difference with girls reporting a healthier food intake than boys, in line with the recommendations. When it comes to energy intakes from sugar, studies have shown diverging results. In a Swedish national survey on food habits among adolescents, ‘Riksmaten’, girls had a higher intake of total energy coming from sugars^(30)^. The present results also indicate a higher intake of sweets and/or snacks among 16-year-old girls compared with boys, as one of the few exceptions to the healthier food choices made by girls. However, we found a lower consumption of SSBs among girls than among boys, in line with other studies^(31,32)^.

To promote a healthy diet, it may be useful to know whether there are any associations between different food intakes among children and adolescents. There is therefore a need for further studies exploring how choices of different foods are associated with each other and to find out whether there are any key foods indicating an overall healthy diet.

## Strengths and weaknesses of the study

Few internationally published studies have described food intake and nutrition time trends among children and adolescents. To use a health questionnaire in preparation for a health dialogue and as a starting point to talk about a child's or adolescent's health has been regarded as useful to start a discussion about lifestyle changes^(21)^. The students are not anonymous in the health dialogue with the school nurse, which contrasts with most health surveys, where participants just respond to a questionnaire without a subsequent health dialogue. This difference may have influenced the answers. It could be a weakness, since the students may have amended their answers in line with what they thought was expected, a process called ‘social desirability bias’, but it could also have enhanced the quality of the answers, as the students had the opportunity to discuss their answers with the school nurse. We know from previous studies that students find it important to be understood by the school nurse and to discuss their health with the school nurse from a person-centred approach^(21)^. Therefore, it can be assumed that students completing this questionnaire want to be truthful in their answers. Another strength of the present study is the small internal dropout and the high percentage of participation. To provide a further context to the setting and study population, the distribution between boys and girls was representative of Swedish children and adolescents. Twenty-seven per cent of the adult population of the county had a higher education (>12 years of education) (compared with 33 % in the whole of Sweden), and 9 % of children and adolescents of the included age groups in the county had a foreign background (compared with 11 % in Sweden)^(22)^. This should be considered when interpreting external validity.

Our choice to measure food intake with a few questions as a food screener is another aspect worth discussing. The questions were part of the ‘My Health’ questionnaire, which was designed to serve as an underlay in a health dialogue about the overall health at the health visit in School Health Services. A few questions would be less burdensome in contrast to a full-length Food Frequency Questionnaire. Other methods, such as observing food habits by means of food diaries, are not suitable for studies with a large number of participants. The question of amount (portion size) is most likely of value^(33,34)^. The present study did not include any questions about the exact amounts of the foods. It is therefore possible that the food screener used in the present study fails to reflect the whole picture. Some students may have eaten more, or less, healthy than it appears from their responses, since a food screener such as this does not capture the amount in detail, and both the amount and the intake frequency could be of importance for health. However, people may find it easier to remember the frequency of their intake than to estimate a quantity, and frequency as a parameter could reflect the diet in an acceptable way for most people.

Another limitation in the present study is that we were unable to follow individuals over time, since we could not link individual data over time. Therefore, the difference between the age groups should not be interpreted at an individual level, although the present results imply a deterioration with age. Also, the healthy time trend that is seen for most of the foods should not be interpreted as an improvement at an individual level.

However, data on a population level from a Swedish report point in the same direction, with increased vegetable consumption and decreased consumption of milk between 2010 and 2015, which strengthens the reliability of the results^(22)^.

## Conclusions

Few children and especially older adolescents reported an overall healthy diet. However, there was a tendency for an improvement in food intake over time, with some exceptions. Eating enough fruit seemed to be the hardest of the food goals to achieve. Finding methods to support all children and especially older adolescents to eat healthier, including eating more fruit, is of great importance to public health.
